# Outbreak of bacterial phlebitis related to peripheral intravenous catheters at a general hospital in Brazil

**DOI:** 10.1186/2047-2994-4-S1-P215

**Published:** 2015-06-16

**Authors:** B Quental, R Saad, L Duarte, J Oliveira, A Frazilio

**Affiliations:** 1 Infectious Diseases, Hospital São Camilo, São Paulo, Brazil; 2 Hospital São Camilo, São Paulo, Brazil

## Introduction

Device related infections are prevalent all over the world. Peripheral intravenous catheters are the most used intravascular devices and the less associated with infections.

## Objectives

To describe the investigation and control measures of an outbreak of bacterial phlebitis at a general hospital in São Paulo, Brazil.

## Methods

Bacterial phlebitis was defined according to the Centers for Disease Control criteria. Each patient’s data were collected from their medical files. After tabulation of the data, we could find common factors and propose control measures.

## Results

From January to June, 2013, 11 cases of bacterial phlebitis related to peripheral Intravenous catheters were reported. We could not find only one factor associated. Most of the cases occurred after 2 days of the punction (42%), and 72% of them were performed at the emergency department. We noticed that 37% of these catheters were manipulated in the ICU and 48% of the punctures were localized at the antecubital fossa. Of these cases, only one patient cursed with bloodstream infection, and this was due to *Staphylococcus aureus* methicillin sensible. We performed observational auditing of insertion and manipulation of these catheters, and we could notice that the most frequent process problems were hand hygiene and hub disinfection. There was no change in the kind of material used in the hospital. Based on these findings, we proposed several measures for infection control, including a hand hygiene campaign, discussion of every case with the multiprofessional team, a global training of 100% of the health care team regarding punction and manipulation of the catheters, reinforcement of daily evaluation of the need of maintaining the catheter and priorization of more distal punction. The outbreak was controlled in august, 2013.

## Conclusion

Active infection control programs, including infection surveillance and implementation of prevention measures are important for all types of intravascular devices, including peripheral Intravenous catheters, in order to improve patient safety.

## Disclosure of interest

None declared.

